# *Saccharomyces cerevisiae* Induces Immune Enhancing and Shapes Gut Microbiota in Social Wasps

**DOI:** 10.3389/fmicb.2019.02320

**Published:** 2019-10-15

**Authors:** Niccolò Meriggi, Monica Di Paola, Francesco Vitali, Damariz Rivero, Federico Cappa, Francesco Turillazzi, Agnese Gori, Leonardo Dapporto, Laura Beani, Stefano Turillazzi, Duccio Cavalieri

**Affiliations:** ^1^Dipartimento di Biologia, Università degli Studi di Firenze, Firenze, Italy; ^2^Istituto di Biologia e Biotecnologia Agraria, Consiglio Nazionale delle Ricerche, Pisa, Italy

**Keywords:** innate immunity, immune training, *Saccharomyces cerevisiae*, gut microbiota, *Polistes dominula*

## Abstract

Trained immunity is the enhanced response of the innate immune system to a secondary infection after an initial encounter with a microorganism. This non-specific response to reinfection is a primitive form of adaptation that has been shown to be conserved from plants to mammals. Insects lack an acquired immune component and rely solely on an innate one, and they have expanded it upon traits of plasticity and adaptation against pathogens in the form of immune priming. The recent discoveries of the role of *Saccharomyces cerevisiae* in the insect’s ecology and the ability of this yeast to induce trained immunity in different organisms suggest that insects could have developed mechanisms of adaptation and immune enhancing. Here, we report that two yeast strains of *S. cerevisiae*, previously shown to induce trained immunity in mammals, enhance resistance to *Escherichia coli* infection in the paper wasp *Polistes dominula*. The reduction of injected *E. coli* load by *S. cerevisiae* strains was statistically significant in future foundresses but not in workers, and this occurs before and after hibernation. We thus investigated if the effect on *E. coli* was mirrored by variation in the gut microbiota composition. Foundresses, showing immune enhancing, had statistically significant changes in composition and diversity of gut bacterial communities but not in the fungal communities. Our results demonstrate that *S. cerevisiae* can prime insect responses against bacterial infections, providing an advantage to future foundress wasps to carry these microorganisms. Understanding the mechanisms involved in the generation of specific and long-lasting immune response against pathogenic infections in insects and the influence of the induction of trained immunity on the commensal gut microbiota could have a major impact on modern immunology.

## Introduction

Immunological memory is an important evolutionary trait that improves host survival upon secondary infection (Netea et al., 2011, 2019). Trained immunity is a newly discovered mechanism of innate immune memory, causing metabolic and epigenetic reprograming of innate immune cells [monocytes/macrophages, natural killer (NK) cells] (Netea et al., 2011, 2019). This non-specific response describes an inflammatory protection against a microorganism upon a second encounter, independently from adaptive immunity, and it has been shown to be present not only in mammals but also in plants (Durrant and Dong, 2004) and insects (Pham et al., 2007; Rodrigues et al., 2010; Norouzitallab et al., 2016). This discovery represents a paradigm change in the biology of immunity with respect to the rigorous division between innate and adaptive immune responses.

Viruses, parasites, and many bacterial or fungal cells or components of their cell wall [lipopolysaccharide (LPS), β-glucan, and chitin] represent strong stimuli of innate immune memory (Mulder et al., 2019). In mice models deficient for functional T and B lymphocytes, β-glucans from fungal cell walls induce trained immunity against non-pathogenic *Candida albicans via* functional reprograming of monocytes, through histone methylation, leading to enhanced cytokine production *in vivo* and *in vitro* (Quintin et al., 2012).

Recent experiments from our group showed that human monocytes stimulated by chitin from *Saccharomyces cerevisiae* lead to enhanced ability to eliminate a wide range of microorganisms, such as *C. albicans* (an opportunistic fungus), *Staphylococcus aureus* (Gram-positive bacterium), or *Escherichia coli* (Gram-negative bacterium) (Rizzetto et al., 2016).

The study of the occurrence of trained immunity in different organisms can be a leading approach to unravel the robustness and plasticity of this phenomenon. An insect model of immune memory is important for a deeper understanding of host defense and thus identifying the most effective approaches to modulate it. Insects evolved a complex innate immune system allowing the rapid elimination of unwanted microorganisms (Rosengaus et al., 1999). Defensive mechanisms against pathogens are mainly related to the activity of phagocytic cells present in the hemolymph and to the production of antimicrobial peptides (Gillespie et al., 1997; Lehrer and Ganz, 1999). Indeed, the activation of innate immunity in vertebrates and invertebrates showed shared mechanisms in the defense against microbial pathogens (Hoffmann et al., 1999), such as the observed activation of nuclear factor (NF)-κB family in Toll-like receptor (TLR) signaling pathways in *Drosophila melanogaster* after fungal interaction (Silverman and Maniatis, 2001). Although lacking elaborate immune-specific reactions based on the “memory” of previously encountered pathogens, as in vertebrates, insects can show an increased resistance upon a secondary exposure to a microorganism (Little and Kraaijeveld, 2004; Sadd and Schmid-Hempel, 2006).

To date, the plasticity of the immune response in invertebrates has been mainly associated with a species-specific response against the pathogen responsible for the primary infection (immune priming) (Milutinović and Kurtz, 2016; Cooper and Eleftherianos, 2017). This immunization can last for days or weeks after the primary infection (Sadd and Schmid-Hempel, 2006) and can be vertically transferred across generation (Sadd et al., 2005; Cooper and Eleftherianos, 2017; Bordoni et al., 2018). Recently, it has been shown that immune priming in *D. melanogaster* is related to the activation of the phagocytes acting as effectors of the response against *E. coli* (Elrod-erickson et al., 2000). This finding suggests that immune priming determines an increase of immune activity with a reduced probability of reinfection through an improved clearance of pathogens. However, many aspects related to trained immunity in invertebrates remain unclear.

Recent reports have shown that the yeast *S. cerevisiae* exploits social wasps as vectors and natural reservoir for winter survival and summer dispersal (Stefanini et al., 2012). *Polistes dominula* paper wasps, in particular, preserve yeasts in their guts and presumably spread them in the environment but also favor hybridization in their intestinal environment, allowing the formation of genetically recombined strains of *S. cerevisiae*, and other yeasts (Stefanini et al., 2016). It is known that gut microbial communities locally influence immune response (Cerf-Bensussan and Gaboriau-Routhiau, 2010; Arnolds and Lozupone, 2016). Less clear is if the yeasts determine physiological responses in the insects, also through modulation of gut microbiota.

According to present knowledge (Stefanini et al., 2012), *S. cerevisiae* is a natural component of paper wasps’ gut microbiota, and it plays a crucial role in the insects’ ecology. The occurrence of a non-pathogenic microorganism in the gut could have the potential to alter the immunocompetence and gut microbial balance of its host. Here, we investigated if two strains of *S. cerevisiae*, previously demonstrated able to induce trained immunity in mammals (Rizzetto et al., 2016), elicit an immunization in *P. dominula* against bacterial infections, and promote gut microbiota modulation. These findings allow us to elucidate the complex relationships between host immunocompetence and its microbiota in the context of wasp natural history.

## Materials and Methods

### Insect Sampling and Housing Maintenance

Wasps of the species *P. dominula* were sampled from the “insect open lab,” an experimental wasp orchard grass located in a field at the university campus in Florence (Sesto Fiorentino, Central Italy, 43°50′7″N, 11°11′46″E). Two different wasp castes, workers (collected before sexual emergence and at the beginning of summer) and future foundresses (female wasps that had not yet found a nest), were considered for immunological trials. The foundresses were tested before and after the winter diapause, a life condition in which different hormonal balance and fluctuation of insect immune system occur (Kubrak et al., 2014).

After sampling, the wasps were pooled and maintained in cohousing under controlled conditions (according to environmental photoperiod and room temperature) in sterile plastic cages and fed with autoclaved water and 40% D-glucose solution, referred to as sugar solution (SS) *ad libitum* for 7 days. After this period, each wasp was randomly assigned to yeast treatment (SS plus yeast) or to a control group (only SS), split, and kept separately in sterile 35-mm Petri dishes for the entire trial period, in order to avoid trophallaxis or any wasp–wasp interactive behavior.

### *S. cerevisiae* and *E. coli* Strain Cultivation

Two *Saccharomyces cerevisiae* strains (YP4 and YH1) belonging to a clinical yeast collection of isolates from human fecal samples (Rizzetto et al., 2016; Ramazzotti et al., 2019) were tested as enhancers of resistance to bacterial infection in paper wasp *P. dominula*.

The two strains were previously proven for their ability to induce training immunity in human cells and mice (Rizzetto et al., 2016) and for different T-polarizing cytokine production in humans (Ramazzotti et al., 2019). These strains also differ in genetic background, as observed by previous analysis of *S. cerevisiae* population structure (Ramazzotti et al., 2019). YP4 strain belongs to the “Human Gut 2” cluster (referred to as HG2), which includes strains isolated from wasps and human intestine, with a common ancestral lineage, whereas YH1 belongs to the HG3 cluster, which includes wasps and human gut strains and other strains isolated from bakery, wine, and other fermentations, descending from a common ancestor, previously identified as West Africa (Liti et al., 2009).

Table 1 summarizes the different genetic, phenotypic, and immunophenotypic characteristics of the two tested strains, as reported by our previous studies (Rizzetto et al., 2016; Ramazzotti et al., 2019).

**TABLE 1:** Summary of genetic, phenotypic, and immunomodulatory traits of the two tested *Saccharomyces cerevisiae* strains.

	***S. cerevisiae* strains**		**References**
		
	**YP4**	**YH1**	
Source	Human feces	Human feces	Ramazzotti et al., 2019
Genetic background (microsatellite loci and whole-genome sequencing)	Human and insect gut (HG2) cluster; HG2 ancestor, and pure lineage	Human and insect gut (HG3) cluster; West Africa ancestor lineage	Liti et al., 2009; Ramazzotti et al., 2019
Phenotypic traits	Low sporulation (<25%)	High sporulation (>25%)	Ramazzotti et al., 2019
	Low invasiveness	Invasive	Ramazzotti et al., 2019
	No pseudohyphal formation	With pseudohyphal formation	Ramazzotti et al., 2019
	Cell wall composition: chitin, 15.84 ± 3.77; glucan, 56.04 ± 5.17; mannan, 28.11 ± 0.43	Cell wall composition: chitin, 12.31 ± 1.68; glucan, 51.06 ± 1.79; mannan, 36.62 ± 8.0	Rizzetto et al., 2016; Ramazzotti et al., 2019
**Trained immunity**			
Human monocytes [test of training with *S. cerevisiae* strain toward lipopolysaccharide (LPS), Pam3Cys4, or *Candida albicans* stimulation; cytokine profiles]	Infection agent: LPS → high level of interleukin (IL)-6 and production tumor necrosis factor (TNF)-α P3C → high level of TNF-α and IL-6 *C. albicans* → high level of IL-6	Infection agent: LPS → IL-6 and TNF-α production P3C → high level of IL-6 and TNF-α production *C. albicans* → not performed	Rizzetto et al., 2016
C57BL/6 mouse model	Chitin training before fungal infection mediates resistance against *C. albicans* systemic infection. Treatment with chitin markedly enhanced survival of infected mice	Not performed	Rizzetto et al., 2016
Immunophenotyping [T-polarizing cytokine levels upon human peripheral blood mononuclear cell (PBMC) challenge]	high IL-17, low interferon (IFN)-g	low IL-17, high IFN-g	Ramazzotti et al., 2019

The *Saccharomyces cerevisiae* strains were grown on yeast peptone dextrose (YPD) broth medium (2% yeast extract, 1% peptone, and 2% D-glucose) at 30°C. Yeast cells were counted with a Bürker chamber to determine the title and washed using sterile phosphate-buffered saline (PBS) solution and then were resuspended in SS and orally administered with sterile tips at a concentration of 10^8^ cells per wasp (Stefanini et al., 2016).

For bacterial infection, non-pathogenic *E. coli*, an immune elicitor commonly used to test immune competence in insects (Cappa et al., 2015; Cini et al., 2018), was used. *E. coli* XL-1 Blue, tetracycline resistant {chromosomal genotype: mutant alleles: recA1 endA1 gyrA96 thi-1 hsdR17 supE44 relA1 lac [F’ proAB lacIqZΔM15 Tn10 (Tetr)]}, was grown on Luria–Bertani (LB) medium (1% tryptone, 0.5% yeast extract, and 1% NaCl), added with tetracycline 10 μg/ml in order to exclude any other microbial contaminants. This microorganism was selected because it is not naturally found in *P. dominula*; thus, we can exclude its presence in our wasps prior to infection.

### Insect Model *P. dominula*: Housing, Feeding, and Infection

In order to observe the effect of administration of *S. cerevisiae* strain on bacterial clearance in an insect model, the first group of autumn foundresses (*N* = 40 pre-hibernation foundresses) was preliminary tested. The wasps were divided into two subgroups: 20 foundresses fed with SS to which yeast strain YP4 was added, while the remaining 20 wasps were fed with SS without any yeast added, as a control group (Supplementary Figure S1).

**FIGURE 1:**
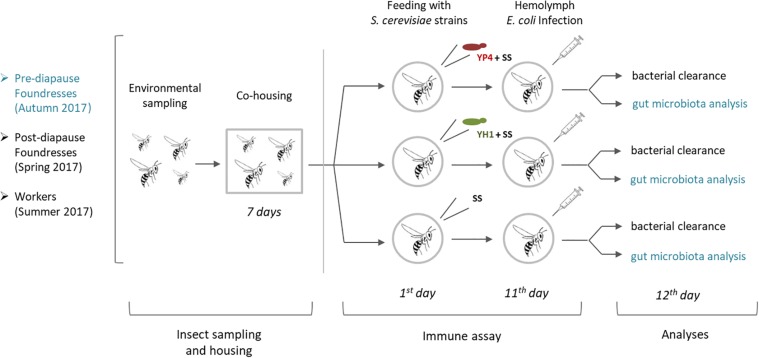
Schematic representation of experimental workflow. After environmental sampling and cohousing for 7 days, *Saccharomyces cerevisiae* strains (YP4 and YH1) were administered in foundresses of *Polistes dominula* wasps collected in different seasons (autumn and spring) and life stages (pre- and post-diapause) and workers collected in summer. At the 11th day, bacterial clearance was compared among treated and control groups. In the pre-diapause foundresses collected in autumn (in blue), gut microbiota analysis was also performed. SS, sterile sugar solution (40% D-glucose). Details were reported in the section “Materials and Methods.”

A second experiment (Figure 1) was conducted with wasps of different castes and in different seasons. A group of autumn foundresses (pre-hibernation foundresses) was divided into three subgroups (*N* = 62): 19 foundresses were fed with SS added with YP4 strain, 20 with SS and YH1 strain, and the remaining 23 with SS without any yeast added (control group). A group of spring foundresses (*N* = 84) was collected after the winter diapause and similarly divided into three subgroups: 31 were fed with SS added with YP4 strain, 33 with SS and YH1 strain, and 20 with SS only.

Workers (*n* = 53) were collected in summer from 10 colonies (at least five workers *per* colony) and treated as follows: 18 workers were fed with SS and YP4 strain, 19 workers with SS and YH1 strain, and 16 workers with SS without yeast (control group).

After feeding, foundresses were maintained at 8°C in the dark and fed *ad libitum* with sterile SS for 10 days (Stefanini et al., 2016). Workers were maintained in similar conditions to foundresses except for temperature, which was kept around 20°C, according to the seasonal temperature in which the workers emerge in the colony. After 11 days of feeding, bacterial infection with *E. coli* was performed. Furthermore, in order to exclude the presence of naturally occurring tetracycline-resistant microorganisms, five wasps were randomly taken from the same collection groups. Their whole body, gut included, was dissected and plated on LB agar, added with tetracycline 10 μg/ml, and incubated for 72 h at 37°C.

*Escherichia coli* cells were grown aerobically in LB medium plus tetracycline 10 μg/ml at 37°C. Cells were counted on a Bürker chamber, washed using PBS solution, and resuspended in PBS. Then, *E. coli* (10^5^ cells) was injected in each wasp with a Hamilton^TM^ micro syringe through the intersegmental membrane between the second and third abdominal tergites (Cini et al., 2018).

After injection, wasps were maintained in the dark at room temperature (20°C) for 24 h. The dead wasps were removed. Each wasp was then dissected under sterile conditions and homogenized in 1 ml of PBS solution using a sterile pestle. Before homogenization, the gut was collected for subsequent metagenomic analysis while the sting and the venom sac were removed in order to avoid possible reduction of the bacterial count due to the presence of antimicrobial peptides in the wasp venom. The homogenate was serially diluted and plated on LB solid medium plus tetracycline 10 μg/ml and then incubated at 37°C overnight.

### Bacterial Clearance Evaluation

The day after infection (12th day), bacterial clearance was evaluated by counting bacterial colony-forming units (CFUs) per milliliter per wasp. We performed the bacterial clearance test as a good proxy of insect immunity, since injection of live bacteria provides an integrative view of the activation of the organismal immune system (Charles and Killian, 2015). Bacterial clearance was compared among wasps belonging to different castes (foundresses and workers), treatments (control and YP4 and YH1 strains), and seasons for foundresses (autumn and spring), by using generalized linear mixed models (GLMMs) where the experiments (three for foundresses and one for workers) were included as a random effect. The effect of fixed factors and their interaction have been tested, obtaining a type III analysis of variance table, by using the Anova function of the *car* R package (Fox and Weisberg, 2019). Moreover, the effect of individual yeast strains compared to the control has been also assessed by using the summary function of the *stat* R package. The generalized linear models (GLMs) have been carried out, grouped and separated for the two experiments as well as for the two castes (foundresses and workers).

### Genomic DNA Extraction From Gut Bacterial and Fungal Communities and Sequencing

The wasp guts were collected and stored in RNAlater Stabilization Solution (Invitrogen, Thermo Fisher Scientific) in sterile microcentrifuge tubes at −20°C until DNA extraction. DNA extraction from gut samples was performed using DNeasy PowerLyzer PowerSoil Kit (QIAGEN) following the manufacturer’s protocol. The total DNA was quantified by a Tecan quantification device (Life Sciences). The sequencing was carried out by an Illumina MiSeq platform (BMR Genomics sequencing service of the University of Padova, Italy^1^). DNA sequencing on bacterial communities was performed on the V3–V4 region of the 16S ribosomal RNA (rRNA) genes by using the primers Pro341F (5′-CCTACGGGNBGCASCAG-3′) and Pro805R (5′-GACTACNVGGGTATCTAATCC-3′) (Takahashi et al., 2014). The same guidelines for fungal communities were carried out on internal transcribed spacer (ITS) 2 rRNA genes by using the primers ITS3 (5′-GCATCGATGAAGAACGCAGC-3′) and ITS4 (5′-TCCTCCGCTTATTGATATGC-3′) (White et al., 1990). Illumina sequencing reads are available at the European Nucleotide Archive^2^ under accession study PRJEB32390.

### Gut Microbiota Analysis

Demultiplexed sample libraries were visually inspected with the FastQC program (Andrews, 2010); low-quality ends of forward and reverse reads were trimmed using Sickle (Joshi and Fass, 2011) with a quality cutoff of 20 and a length threshold after trimming of 200. MICCA pipeline v. 1.7.2 (Albanese et al., 2015) was used for operational taxonomic unit (OTU)/sequence variant (SV) picking as follows: forward and reverse reads were joined with *micca mergepairs* command, and reads with N bases were discarded with command *micca filter.* OTU/SV picking and chimera checking were performed with the *miccaotu* command and the UNOISE3 protocol as a picking algorithm. Taxonomy was assigned to the representative sequences of the identified OTUs/SVs classified using the RDP classifier v. 2.11 (Wang et al., 2007).

Subsequent analyses were performed in R (v.3.42; R Core Team, 2018), employing package *phyloseq* v.1.22.3 (McMurdie and Holmes, 2013) to import data [as biological observation matrix (BIOM) files], to perform PCoA ordination analysis (using the Bray–Curtis distance measure), and to plot microbiota composition as a bar plot. Alpha diversity indices were calculated with the *microbiome* package (Lahti et al., 2017), and overall differences were tested with ANOVA, while species accumulation curves were calculated with the *ranacapa* package (Kandlikar et al., 2018). Prior to any analysis, count data were scaled with CSS transform as implemented in the *metagenomeSeq* (Paulson et al., 2013). Heatmaps reporting OTU distribution in different samples were created with the *pheatmap* package (Kolde, 2012). For heatmap construction, rows (i.e., OTUs/SVs) were ordered based on the results of the *plot_heatmap* command in the *phyloseq* package, using PCoA as the ordination method and Bray–Curtis as the distance measure, while columns (i.e., samples) were ordered based on Euclidean distance (as implemented in the *pheatmap* package). Color maps of OTU/SV abundance were scaled on the rows. Association between gut microbial community diversity and bacterial CFUs found in the hemocele post infection (a proxy for immune response priming and activation) were evaluated with distance-based permutational multivariate analysis of variance (PERMANOVA; Bray–Curtis distance; 9,999 permutations) using the *adonis* function in the *vegan* v.2.5-2 (Oksanen et al., 2018) package, as well as employing multivariate-structure-based GLMs (negative binomial), as implemented by the *manyglm* command in the package *mvabund* (Wang et al., 2012). STAMP (Parks et al., 2014) software was used to compare relative abundance of OTUs at different taxonomic levels and to create plots. We used Welch’s *t* test (with no correction for multiple comparison) and displayed only OTUs with a *p* < 0.05.

## Results

### *S. cerevisiae* Strains, Able to Induce Trained Immunity in Mammals, Enhance Bacterial Clearance in Foundresses of Social Wasp *P. dominula*

In order to test the ability of *S. cerevisiae*, a generally recognized as safe (GRAS) yeast, to enhance resistance to bacterial infection, we selected two *S. cerevisiae* strains based on their previously observed ability to induce training immunity in human cells and mice (Rizzetto et al., 2016).

**FIGURE 2:**
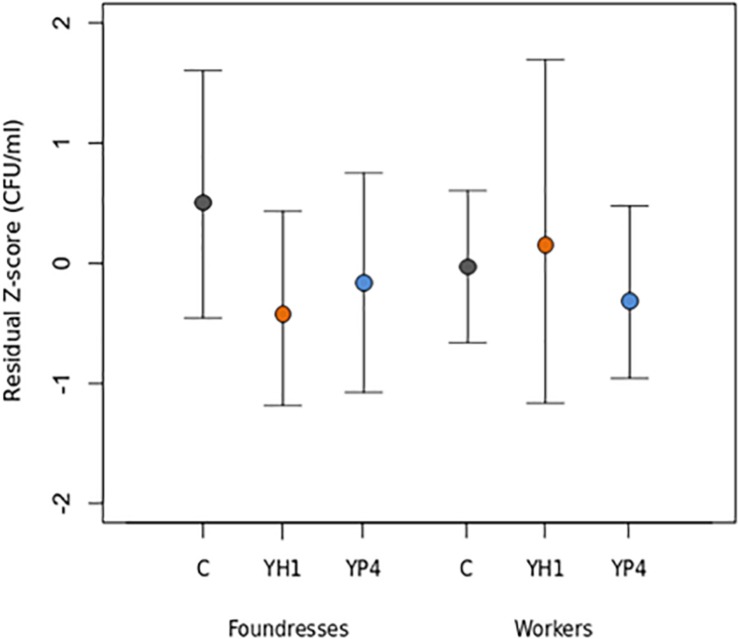
Bacterial clearance in *P. dominula* in foundresses and workers after yeast administration. Mean and standard deviation of residual load of *Escherichia coli* colony-forming units (CFUs) per milliliter in control (C) and treated groups (YH1 strain and YP4 strain administration) in foundresses and workers, at 24 h after bacterial infection. Data are representative of the bacterial clearance measure, reported as *z*-score transformation (residual *z*-score of CFUs per milliliter).

In a first experiment, bacterial clearance was preliminarily tested on one group of autumn foundresses after administration of the YP4 yeast strain (Supplementary Figure S1). Subsequently, an immune trial was performed on two different wasp castes (future foundresses and workers) in three different seasons (summer workers and autumn and winter foundresses, see section “Materials and Methods”). The experimental design is depicted in Figure 1. By immune trials performed on foundress wasps, we observed that the bacterial clearance was lower in the control group than in the treated groups (Figure 2, Supplementary Datasheet S2, and Supplementary Figure S4). Firstly, data of the bacterial clearance from the two different experiments (including the preliminary test with the sole YP4 strain administration, as reported in Supplementary Figure S1) were analyzed separately, showing a statistically significant effect of yeast treatment on the reduction of *E. coli* load (Table 2). Then, we have extended the analysis to all groups by integrating all variables (treatments, caste state, and season) into a GLMM (Table 3; see section “Materials and Methods”). The GLMM showed that the treatment with *S. cerevisiae* decreased the infection burden (measured as *E. coli* CFUs per milliliter as reported in Table 3 and shown in Figure 2). The treatment effect was significant in the reduction of *E. coli* load regardless the two different trials, also when the foundresses from the preliminary experiment were added in an overall analysis (Table 3).

**TABLE 2:** ANOVA of deviance for 217 total observations (173 foundresses and 44 workers).

	**Variables**	**df**	**χ^2^**	***P***
Preliminary immune trial in foundresses (YP4 strain)	Treatment	1	5,336	0.027
Complete immune trial in foundresses and workers (YP4 vs. YH1 strains)	Treatment	2	10,096	0.006
	Caste	1	2,585	0.108
	Treatment ^∗^ Caste	2	5,547	0.060
Both preliminary and complete immune trials in foundresses and workers (YP4 vs. YH1 strains)	Treatment	2	16,485	<0.001
	Caste	1	2,572	0.109
	Treatment ^∗^ Caste	2	5,563	0.062
Foundresses	Treatment	2	14,233	<0.001
	Season	1	228,375	<0.001
	Treatment ^∗^ Season	2	2,714	0.257
Workers	Treatment	2	1,099	0.577

*Data are separated for preliminary test (only autumn foundresses and YP4 yeast strain), the complete test (treatment and caste after YP4 and YH1 yeast strain administration), and both preliminary and complete immune trials. Separated analyses among foundresses and workers are also reported. df, degree freedom; *P*, *p*-value.*

**TABLE 3:** Generalized linear mixed model (GLMM) analysis.

	**Variables**	**Estimate**	**Standard error**	***Z* Values**	***P***
Complete immune trial in foundresses and workers (YP4 vs. YH1 strains)	YH1-C	–0.511	0.179	–2.86	0.004
	YP4-C	–0.374	0.183	–2.04	0.041
	Caste	−2,894	1.800	–1.61	0.108
	YH1 ^∗^ Caste	0.815	0.345	2.34	0.019
	YP4 ^∗^ Caste	0.079	0.350	0.23	0.820
Foundresses	YH1-C	–0.425	0.213	–2.00	0.046
	YP4-C	–0.490	0.161	–3.04	0.002
	Season	3,424	0.227	15.11	<0.001
	YH1 ^∗^ Season	–0.083	0.303	–0.27	0.785
	YP4 ^∗^ Season	0.312	0.271	1.15	0.251
Workers	YH1-C	0.304	0.404	0.75	0.45
	YP4-C	–0.295	0.404	–0.73	0.46

*Comparison of single variables (combination of treatment, caste, and season) by using GLMM, on bacterial clearance observed in all tested foundresses and workers.*

The experiments and analyses have been repeated for foundresses and workers separately (Table 3), revealing a caste-dependent effect, statistically significant in foundresses but not in workers (Figure 2). Furthermore, we showed a significant interaction effect for the sole strain YH1 (*p* = 0.019, Table 3) that increased bacterial clearance in foundresses but decreased resistance to the infection in workers (higher CFU-per-milliliter counts; Figure 2).

The experiments on the foundresses were performed in autumn (entry into diapause) and spring (exit to the diapause). We found that the levels of CFUs per milliliter were significantly higher in the spring than in the autumn (*p* < 0.001; Table 3). However, the treatment affects the CFU-per-milliliter levels regardless of the season, as shown by the lack of significance of the interaction effect in Table 3 (*p* = 0.785 for YH1 ^∗^ season and *p* = 0.251 for YP4 ^∗^ season).

### Bacterial Clearance Induced by *S. cerevisiae* Treatment Is Associated to Shaping of Gut Microbiota Composition

In order to evaluate the effect of *S. cerevisiae* administration on the gut microbial communities, either via direct interaction or as a consequence of the immune training, we performed characterization of gut microbiota on the autumn foundress group, in which the effect of yeast administration on bacterial clearance was more evident. Autumn was also preferred because it is the season with a higher isolation rate of *S. cerevisiae* strains from wasp guts (Stefanini et al., 2012).

We selected nine gut samples of foundresses per group (treatments and control), pooling them together in a total of three gut samples per group, as representative gut microbiota collection of all tested samples of the respective groups (Supplementary Figure S2). The criteria for sample selection were in accordance with the coefficient-of-variation (CV) analysis calculated by comparison of intragroup and intergroup (intragroup CV%: control group = 15.4; YP4-treated group = 38.2; YH1-treated group = 36.2; intergroup CV%: control vs. YP4 = 46.9; control vs. YH1 = 48; as reported in Supplementary Table S1).

By 16S rRNA V3–V4 region sequencing, a total of 214,089 sequence reads were obtained after quality filtering, clustering in a total of 495 OTUs. The number of 16S rRNA sequences per sample ranged from 1,607 to 51,340. Although the control group showed a low amount of sequence reads, no statistical differences in read number were observed among groups (Supplementary Figure S5).

**FIGURE 3:**
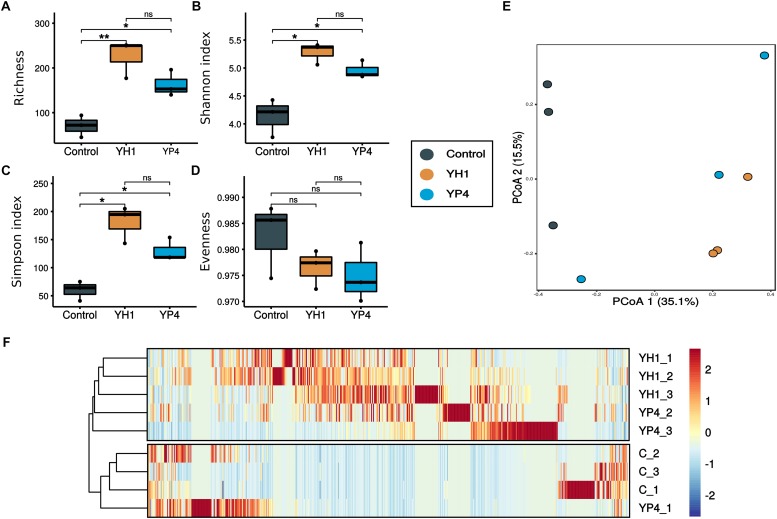
Alpha and beta diversity of gut microbial communities. **(A–D)** Alpha diversity measures: **(A)** richness, **(B)** Shannon diversity index, **(C)** Simpson index, and **(D)** evenness of control and treated (YH1 and YP4) groups. Statistically significant comparisons by *t* test (^∗^*p* < 0.05, ^∗∗^*p* < 0.01, and ns = not statistically significant). **(E)** Principal coordinate analysis (PCoA) ordination based on the Bray–Curtis distance. **(F)** Heatmap reported the frequency of operational taxonomic unit (OTU) abundance within treatment and control groups.

By comparison of the alpha diversity indices among the three groups (control and YH1- and YP4-treated groups), we observed a reduction in richness (Figure 3A) and diversity (i.e., Shannon and inverse Simpson indices; Figures 3B,C) of gut bacterial communities in the control group, with respect to the two treated groups (Figure 3; ANOVA test; richness: *F* = 16.91, *p* = 0.0034; Shannon’s index: *F* = 17.6, *p* = 0.0031; inverse Simpson index: *F* = 18.32, *p* = 0.0027; a pairwise *t* test is reported for every comparison in Figure 3). Moreover, as indicated by a higher evenness index, the microbial community of the control group had more homogeneous abundance distribution than the treated groups (Figure 3D).

PCoA ordination analysis based on Bray–Curtis distances showed a clear separation of gut samples, along the first ordination axis (explaining 35.1% of the variability), according to the type of treatment (Figure 3E). In particular, samples from the YH1-treated group showed a clear separation and a more defined grouping than did the control and YP4 groups (Figure 3E). PERMANOVA was used to test the different distributions of samples, as observed in ordination analysis (Supplementary Table S2). Statistical analysis showed that *S. cerevisiae* treatment has an effect, as a whole, on microbiota composition, when compared to the control group (control vs. treatment: *R*^2^ = 0.3, *p* = 0.01), and the treatments with the two *S. cerevisiae* strains differentially affect the microbiota composition (control vs. YH1 treatment vs. YP4 treatment: *R*^2^ = 0.4, *p* = 0.03). In Figure 3F, the heatmap displays the OTU frequencies and abundance distribution among control and treated samples. We observed different trends of OTU pattern distribution between control and treated groups (separated branches in hierarchical clustering as reported in Figure 3F), as well as between the two different yeast-treated groups.

Generalized linear models analysis (*mvabund* R package) was performed in order to assess the multivariate association between residual CFUs per milliliter (following the bacterial clearance) and gut bacterial community composition, showing significant association (likelihood ratio statistic: 857.4, *p* = 0.002). We found 75 OTUs (Supplementary Datasheet S1) with a significant univariate association to (log)CFUs per milliliter of bacterial infectious agent, regardless of treatments or control. When correction for multiple comparison was applied, *Campylobacter* was still significantly associated to the count of infectious agent (adjusted *p* = 0.013; Supplementary Datasheet S1).

**FIGURE 4:**
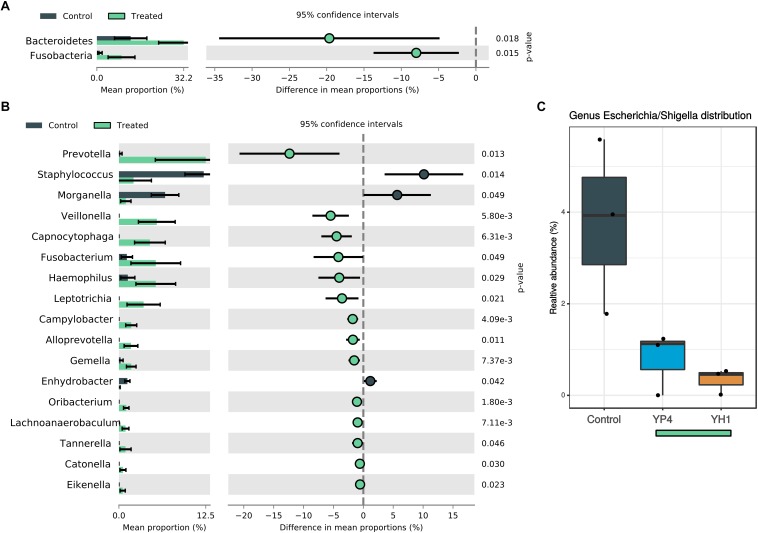
Comparison of statistically significant taxa in treated and control groups. Mean proportion and confidence intervals (%) in control vs. treated (YH1 and YP4) groups explored at phylum **(A)** and genus **(B)** levels (*p*-values by Welch’s *t* test). **(C)** Relative abundance (%) of *Escherichia*/*Shigella* genus distribution between control and treated groups (ANOVA *p* = 0.025).

To evaluate the overall effect of *S. cerevisiae* administration and the subsequent resistance to *E. coli* infection on the wasp gut microbiota composition, we compared the microbial community’s relative abundance (at phylum and genus levels) among treated and control groups, as shown in Figure 4 and Supplementary Figure S3. At the phylum level, an increase of Bacteroidetes and Fusobacteria was observed in treated groups (Figure 4A). At the genus level, 17 genera showed significant different abundances between control and treatment groups (Figure 4B; Welch’s *t* test). Among them, *Staphylococcus* (Firmicutes phylum) and *Morganella* (Proteobacteria phylum) were significantly enriched in the control group. Interestingly, *Escherichia*/*Shigella* genus was significantly enriched in the gut community of the controls compared to the treated groups (Figure 4C; ANOVA *p* = 0.025).

In treated groups, *Prevotella* (Bacteroidetes), *Veillonella* (Firmicutes), *Capnocytophaga* (Bacteroidetes), *Fusobacterium* (Fusobacteria), *Haemophilus* (Proteobacteria), and *Leptotrichia* (Fusobacteria) were found to be significantly abundant (Figure 4B; Welch’s *t* test).

**FIGURE 5:**
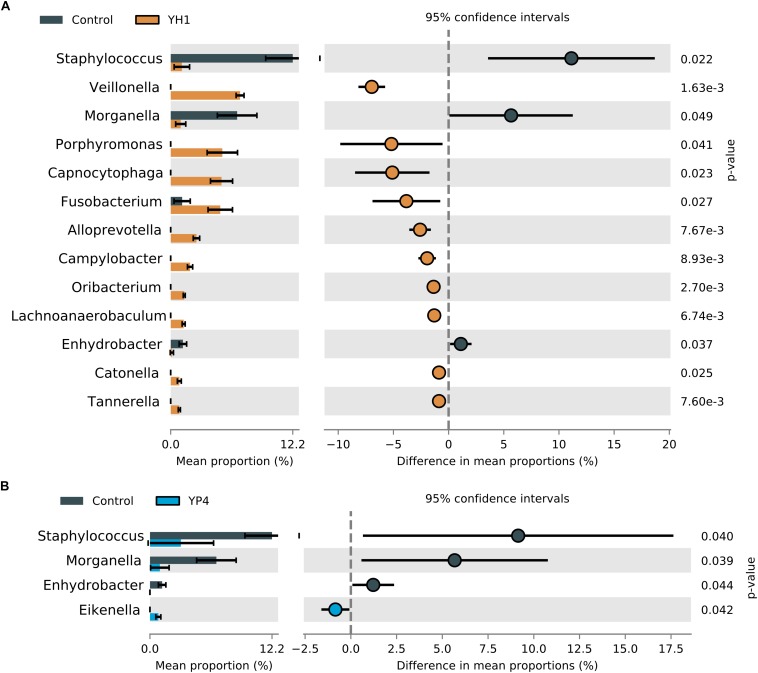
Comparison of statistically significant taxa in YH1- and YP4-treated and control groups. Mean proportion and confidence intervals (%) in **(A)** control vs. treated YH1 and **(B)** control vs. YP4, explored at genus level (*p*-values by Welch’s *t* test).

The two yeast strains highly differed in their ability to change the microbial profiles (Figure 5). While administration of both *S. cerevisiae* strains reduced the levels of *Staphylococcus* and *Morganella*, when compared to the control group (Figures 5A,B), the single comparison between strain administration and control (YH1 vs. control in Figure 5A and YP4 vs. control in Figure 5B) showed a strain-dependent effect on gut microbiota composition. To note, YH1 treatment (Figure 5A) was associated to an enrichment of a higher number of bacterial genera, such as *Fusobacterium*, *Veillonella*, *Alloprevotella*, *Capnocytophaga*, *Porphyromonas*, and *Campylobacter*, compared to YP4 treatment (Figure 5B; Welch’s *t* test).

### *S. cerevisiae* Treatment Does Not Affect the Gut Fungal Communities

**FIGURE 6:**
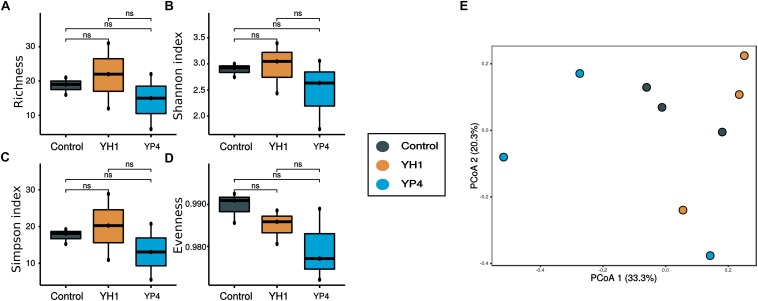
Alpha and beta diversity of gut mycobiota. **(A–D)** Comparison of alpha diversity measures: **(A)** richness, **(B)** Shannon diversity index, **(C)** Simpson index, and **(D)** evenness among treated (YH1 and YP4) and control groups. Statistically significant comparisons by *t* test (^∗^*p* < 0.05, ^∗∗^*p* < 0.01, and ns = not statistically significant). **(E)** Principal coordinate analysis (PCoA) ordination based on the Bray–Curtis distance among fungal communities between control and treated groups.

**FIGURE 7:**
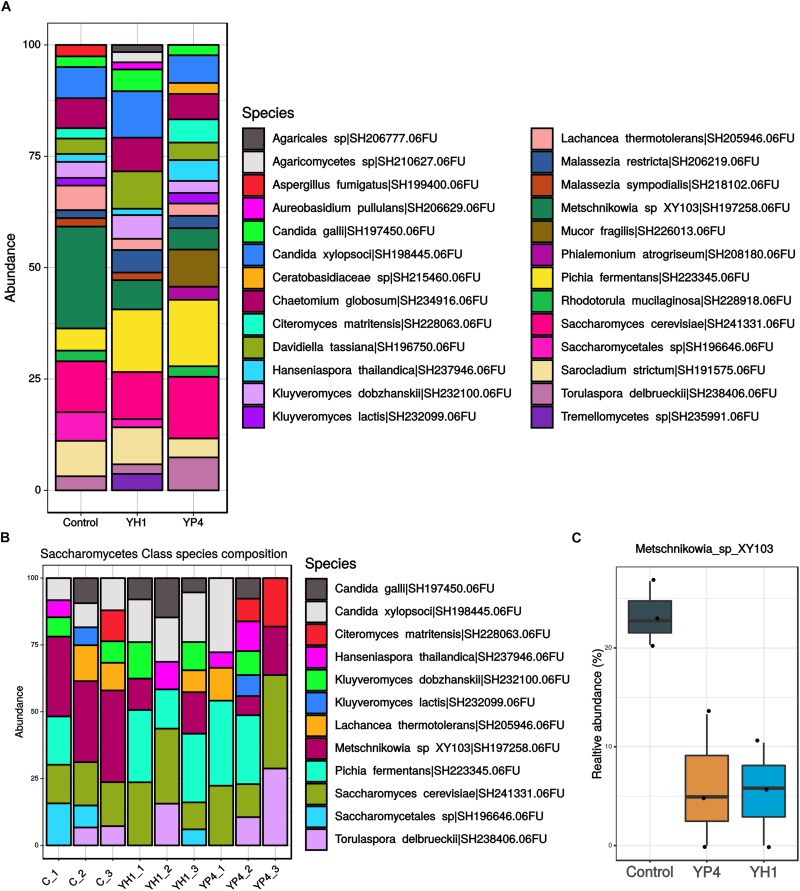
Gut mycobiota community abundance. **(A)** Comparison of gut mycobiota composition in treated and control groups. Bar plot showed the taxa with >1% of relative abundance annotated at species level. **(B)** Focus on *Saccharomycetes* fungal class composition reporting species taxa with >1% of relative abundance. **(C)** Relative abundance (%) of *Metschnikowia* sp. distribution between control and treated groups.

For the fungal ITS2 region sequencing, we obtained a total of 14,646 sequences after quality filtering, clustering in a total of 63 OTUs. The number of sequence reads per sample ranged from 202 to 3,024. Contrary to the gut bacterial community, the mycobiota composition did not show significant differences in richness and biodiversity between control and treated groups, as observed by alpha diversity and PCoA ordination analysis based on the Bray–Curtis distance (Figure 6). Accordingly, PERMANOVA analysis used to test the differences in fungal community composition among groups showed no effect of treatment on gut mycobiota (Supplementary Table S3). In Figure 7A, we reported an overview of relative abundances of fungal taxa (>1%) annotated at the species level in the three groups. No significant association between fungal community composition and bacterial infection load (CFUs per milliliter of *E. coli*) have been observed. The fungal community abundances (Figure 7A) appeared unaffected by the *S. cerevisiae* administration and by bacterial infection in hemocele. Only *Metschnikowia* sp. was found significantly enriched in the control group compared to both treated groups (Figures 7B,C; ANOVA *p* < 0.001).

## Discussion

In recent years, the physiological and ecological relationships between hymenopterans, in particular social wasps, and the yeast *S. cerevisiae* have become evident (Stefanini, 2018). The wasps likely act as natural vectors, hosting and transporting *S. cerevisiae* between different environmental niches and favoring mating among strains (Stefanini et al., 2012, 2016; Ramazzotti et al., 2019). While the advantage provided to the yeast is well understood, the benefit for the wasp to carry *S. cerevisiae* remains unclear. In this study, we tested the hypothesis that *S. cerevisiae* could help the insect to resist infections during hibernation. This hypothesis is supported by previous studies (Quintin et al., 2012; Rizzetto et al., 2016; Rusek et al., 2018; Mulder et al., 2019) showing that fungal cells or components of cell wall (i.e., chitin) are potent stimuli of innate immune memory in mammals. To this aim, we investigated whether two well-characterized *S. cerevisiae* strains, by genetic, phenotypic, and immunophenotypic assays (Ramazzotti et al., 2019), previously proved to induce trained immunity in mammals Rizzetto et al. (2016), are also able to enhance resistance of the invertebrate immune system against bacterial infection and shape the gut microbial communities. A few studies have focused on the nature of the insect immune system, revealing that it can be efficiently primed upon exposure to microorganisms, acquiring a protective effect upon subsequent challenge with the same, and/or different microbes (Cooper and Eleftherianos, 2017).

We used the social wasp *P. dominula* as an animal model for innate immune trials. They evolved mechanisms to tolerate and benefit from the presence of this GRAS microorganism, providing protection against pathogens and maintaining health status, through the development and boosting of the immune system and providing protection against pathogens. Our results showed that *S. cerevisiae* induces in foundresses, but not in workers, a higher response against bacterial infection compared to the controls, possibly *via* the activation of strain-dependent innate immunization. In fact, following YH1 administration when compared to YP4 strain, we observed significant differences in immune enhancing, as well as in the changes of the gut microbiota.

The data presented here, alongside the immune training induced by the same yeast strains in mouse models and human monocytes (Rizzetto et al., 2016), show that the yeast strains can provide protective mechanisms shared between different subphyla, potentially through analog immune training mechanisms occurring in mammals, and insects. The yeast-mediated protection against pathogens could be important to protect hibernating foundresses during overwintering, simultaneously providing yeasts with the environmental niche where they mate and survive, thus suggesting the existence of a complex symbiotic interaction. Complex communication systems have been evolved between the host immune system and the intestinal microbiota (Belkaid and Hand, 2014). Evidence is mounting in support of a dominant and decisive role of gut in shaping and modulating immune responses in the prevention of disease (Hand, 2016). However, the effect of trained immunity on commensal microbial communities, that is, the microbiota, has not been shown yet. The gut microbiota plays a fundamental role in the education and functional tuning of the host immune system, thereby acting as adjuvant to the host immune system and continually driving the nature of immune responses, providing protection against pathogens. In turn, the host immune system has evolved multiple means by which to maintain its symbiotic relationship with the microbiota (Dethlefsen et al., 2007; Cho and Blaser, 2012; Lynch and Pedersen, 2016; Gupta et al., 2018).

Hymenoptera’s gut contains a relatively simplified microbiota, with lesser microbial species as compared to that of mammals, yet social insects harbor microbial communities with highly specialized and beneficial functions in nutrition, protection from parasites and pathogens, and modulation of immune responses (Engel and Moran, 2013).

In both yeast-fed groups, we also observed reduction in potential pathogenic bacterial genera, such as *Staphylococcus*, *Morganella*, and *Escherichia*/*Shigella*, compared to the control group. The observed reduction of *Staphylococcus* load, following yeast feeding, may be also associated with the ability of *S. cerevisiae* to counteract and inhibit *Staphylococcus* biofilm making, as previously observed (Walencka et al., 2007).

The two yeast strains showed different abilities to change the bacterial microbiota composition. The YH1 strain displays the strongest caste-dependent immunomodulatory effect, to the detriment of the resistance to *E. coli* infection in the workers, and shows increased ability in changing the gut microbiota, suggesting that the changes in the bacterial communities are related to strain-specific immunomodulation. While this effect is evident for bacterial communities, *S. cerevisiae* administration does not alter the diversity of the fungal communities. In the latter, only the *Metschnikowia* genus was reduced. Yeast–yeast competition for nutrients are well-known, especially regarding *S. cerevisiae* and *Metschnikowia* growing in fermentative processes (Alexandre et al., 2004; Fleet, 2008; Albergaria and Arneborg, 2016; Ciani et al., 2016; Sadoudi et al., 2017). Our results suggest that the competition between these two yeasts is not limited to grape must but may occur also in the insect gut.

It is unlikely that the observed effects are influenced by a role of *S. cerevisiae* as nutrient supplementation: (i) it is known that in *P. dominula*, the green fluorescent protein (GFP)-labeled *S. cerevisiae*, ingested by wasps, populate the gut for at least 4 months (Stefanini et al., 2012, 2016) (thus, the microorganism is still alive after treatment and survives after passage through the wasps’ digestive tract); (ii) immune training occurred only in foundresses and not in workers, showing a caste-dependent effect in insects that received the same amount of *S. cerevisiae* cells (if the effect would have been related to nutrition, one would expect this to be equal regardless of the caste); (iii) the two strains showed significant differences in the ability to improve the bacterial clearance (a strain-dependent immune-mediated event excludes a general nutritional effect of the yeast); and (iv) the differences in immune enhancing are mirrored by differences in the changes of the microbiota composition (this finding corroborates our conclusion that the effect is mediated by immune responses and not just by feeding).

Overall, this study provides a preliminary indication of the complex interactions existing between insects’ immune responses and their symbiotic gut communities. Future studies using insects as models promise stimulating information that will potentially uncover the relationship between immune priming and physiological functions, as well as their effect on gut microbiota. Our results suggest that yeasts and social insects possess key synergistic elements and that a deeper understanding of these interactions, including the molecular determinants of the priming and trained immunity, may provide significant insights on the host immune system–microbe evolution.

## Data Availability Statement

Data are available as electronic Supplementary Material. Illumina sequencing data are available at the European Nucleotide Archive (http://www.ebi.ac.uk/ena/data/view/PRJEB32390) under accession study PRJEB32390.

## Author Contributions

DC, ST, and NM conceived the study. NM, DR, FC, FT, and AG carried out the experiment. LD and NM performed the statistical analyses. FV performed the metagenomic and data analysis. NM, MP, LB, and DC drafted the manuscript. All authors critically revised the manuscript, approved the final version of the manuscript, and agreed to be held accountable for the content therein.

## Conflict of Interest

The authors declare that the research was conducted in the absence of any commercial or financial relationships that could be construed as a potential conflict of interest.

## Footnotes

1 https://www.bmr-genomics.it/

2 http://www.ebi.ac.uk/ena/data/view/PRJEB32390
