# Supplementary material for: Saccharomyces cerevisiae Induces Immune Enhancing and Shapes Gut Microbiota in Social Wasps
Source: Front Microbiol. 2019 Oct 15;10:2320. doi: 10.3389/fmicb.2019.02320 (PMC6803456; doi:10.3389/fmicb.2019.02320)
Supplement: Supplementary file 1 [file Data_Sheet_1.pdf]

Supplementary Material

Saccharomyces cerevisiae induces immune enhancing and shapes gut microbiota in social wasps

Niccolò Meriggi, Monica Di Paola, Francesco Vitali, Damariz Rivero, Federico Cappa, Francesco Turillazzi, Leonardo Dapporto, Laura Beani, Stefano Turillazzi, Duccio Cavalieri.

Supplementary Material contains:

Supplementary Tables S1, S2, S3

Supplementary Figures S1, S2, S3, S4, S5

Supplementary Datasheet S1, S2

Supplementary Tables

Supplementary Table S1. Bacterial clearance and coefficient of variation analysis used to compare and select gut sample for microbiota analysis.

Gut samples	Bacterial clearance (CFU/ml)			Sum	Average	Standard deviation	Intra-group average	Intra-group average of St. dev.	Intra-groups coefficient of variation (%)	Inter-groups average (C vs YP4)	Inter-groups average (C vs YH1)	Inter-groups St. dev.	Inter-groups coefficient of variation (%)
C.1	17400	20100	21200	58700	19566.67	1955.33							
C.2	17900	20400	26000	64300	21433.33	4147.69	21088.89	3250.13	15.412			7012.27	46.87
C.3	19500	20900	26400	66800	22266.67	3647.37							
YP4.1	6000	8700	11000	25700	8566.667	2502.67							
YP4.2	6200	9600	14600	30400	10133.33	4225.32	8833.33	3377.46	38.24	14961.11		7103.21	48.05
YP4.3	4800	7100	11500	23400	7800	3404.41							
YH1.1	6100	7100	10300	23500	7833.333	2193.93							
YH1.2	6200	7000	12100	25300	8433.333	3200.52	8477.78	3070.65	36.22		14783.33		
YH1.3	6300	7700	13500	27500	9166.667	3817.50							

Supplementary Table S2. PERMANOVA analysis on bacterial communities between each group (Control vs YH1 vs YP4) and within treated and control groups.

	Df	Sums Sqs	Mean Sqs	F model	R2	P value
Group	2	0.893	0.447	1.79	0.374	**0.03**
Residual	6	1.497	0.249		0.626	
Total	8	2.390			1.000	
	**Df**	**Sums Sqs**	**Mean Sqs**	**F model**	**R2**	**P-value**
Treatment	1	0.670	0.670	2.729	0.280	**0.01**
Residual	7	1.720	0.246		0.719	
Total	8	2.390			1.000	

Supplementary Table S3. PERMANOVA analysis of fungal communities between each group (Control vs YH1 vs YP4) and within treated and control groups.

	Df	Sums Sqs	Mean Sqs	F model	R2	P-value
Group	2	0.480	0.240	1.329	0.307	0.1579
Residuals	6	1.085	0.181		0.693	
Total	8	1.565			1.000	
	**Df**	**Sums Sqs**	**Mean Sqs**	**F model**	**R2**	**P-value**
Treatment	1	0.168	0.168	0.843	0.107	0.6391
Residuals	7	1.397	0.200		0.892	
Total	8	1.565			1.000	

Supplementary Figures

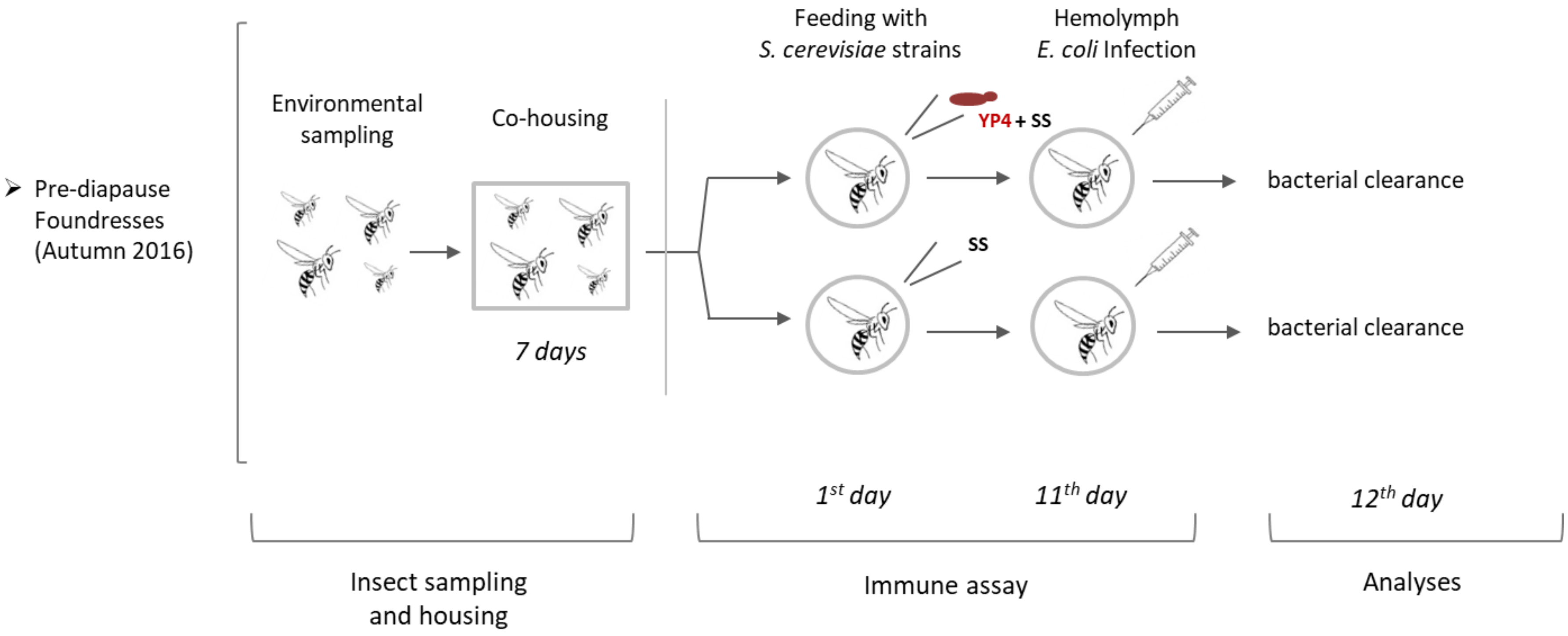

Supplementary Figure 1. **Schematic representation of the preliminary immune trial.** After environmental sampling and cohousing for 7 days of autumn foundresses wasps, *S. cerevisiae* YP4 strain was administrated for 10 days. At 11th day, *E. coli* was injected in hemolymph of wasps and subsequently (at 12th day) bacterial clearance was compared between treated and control group. Note: SS = sterile Sugar Solution (40% D-glucose). Details were reported in Materials and Methods.

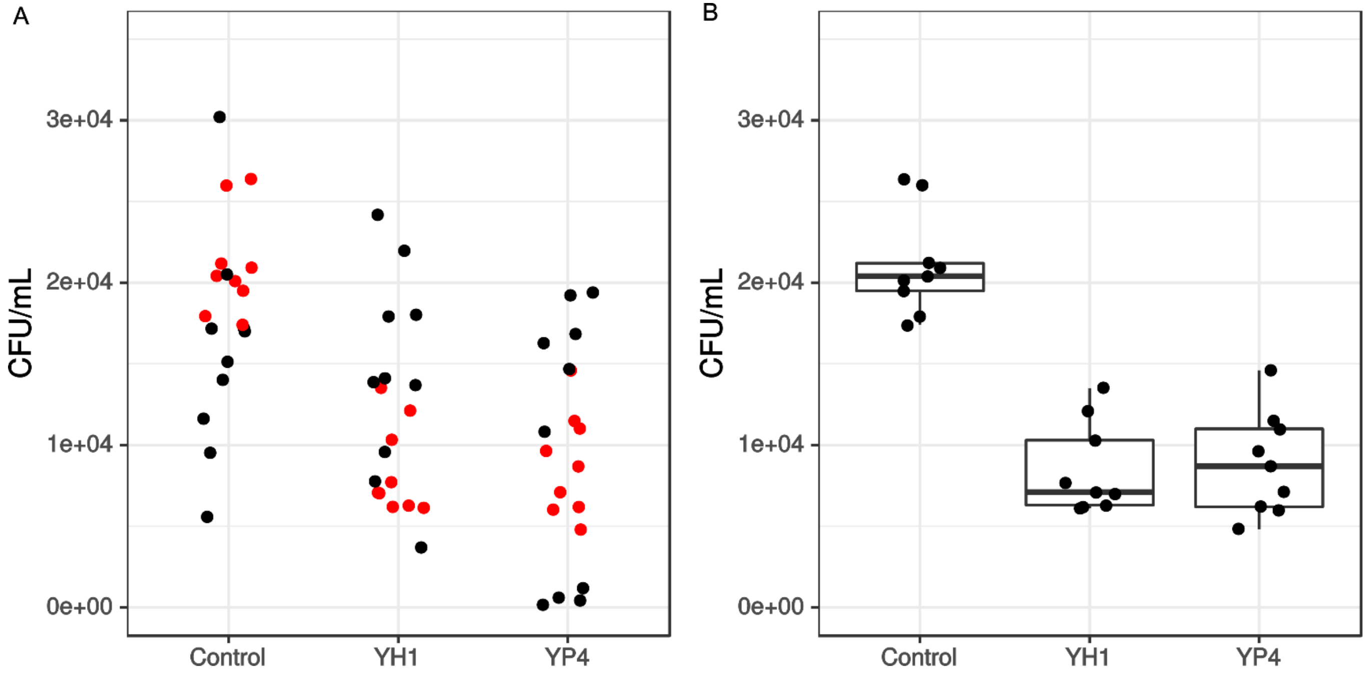

Supplementary Figure 2. **Selection of samples for gut microbiota analysis.** A) Jitter plot reported CFU/ml in control and treated groups from foundresses wasps tested in autumn 2017. Red dots represent the wasp samples selected for gut microbiota analysis. These samples were pooled (three gut per sample) before extraction of genomic DNA of the gut microbial communities. B) Boxplot showed the distribution in terms of CFU/ml of the nine selected gut samples for each control and treatment group.

The criteria used for the gut collection were reported as follow: (i) Hypothesizing that samples with same level of bacterial infection (CFU/ml) could have a same effect on gut microbiota composition, we select gut samples from control group with higher level of bacterial clearance (CFU/ml) with respect to the treatment groups. (ii) Coefficient of Variation intra-group must be lower compared to the Coefficient of Variation inter-groups, as reported in Supplementary Table S1.

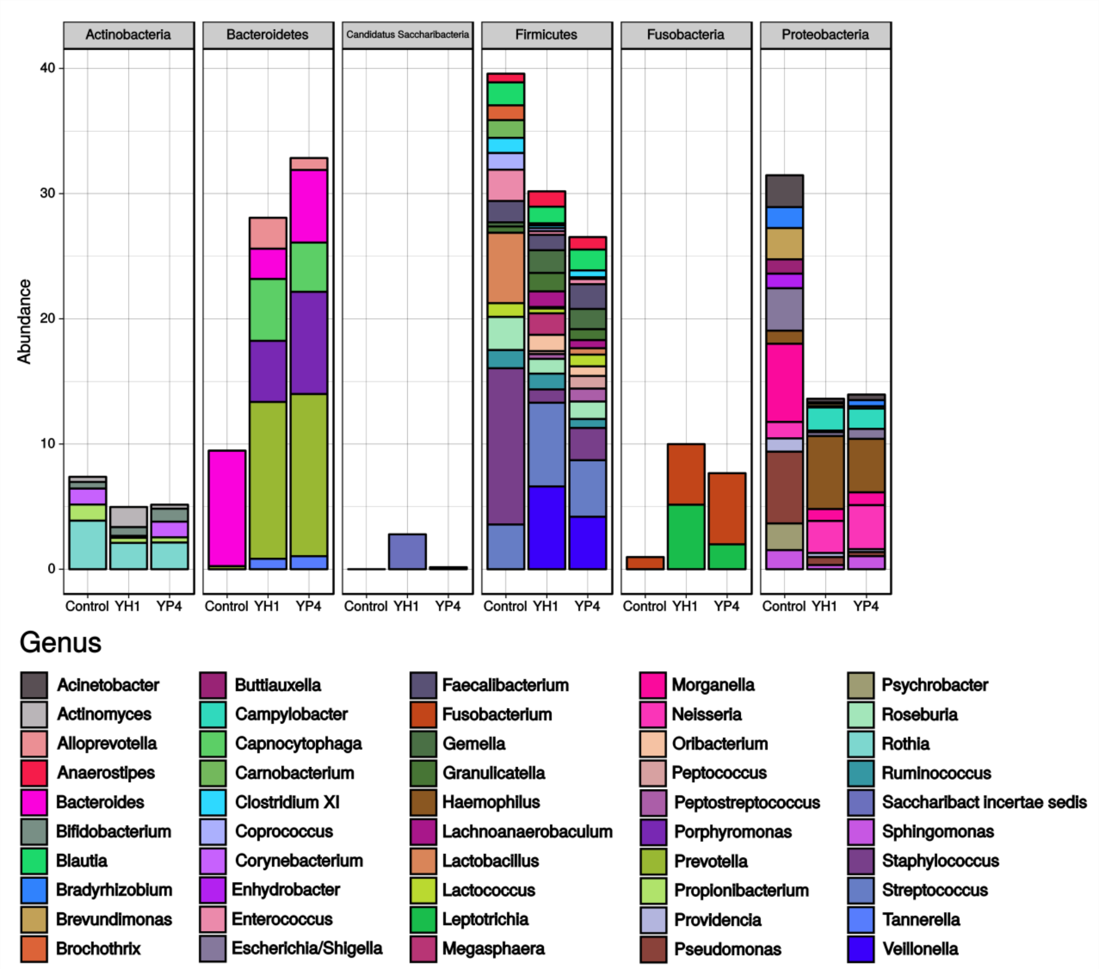

Supplementary Figure 3. **Relative abundance at genus level of gut microbiota composition in treated compared to the control groups.** Barplot showed bacterial genera with >1% of relative abundance grouped at phylum level (columns).

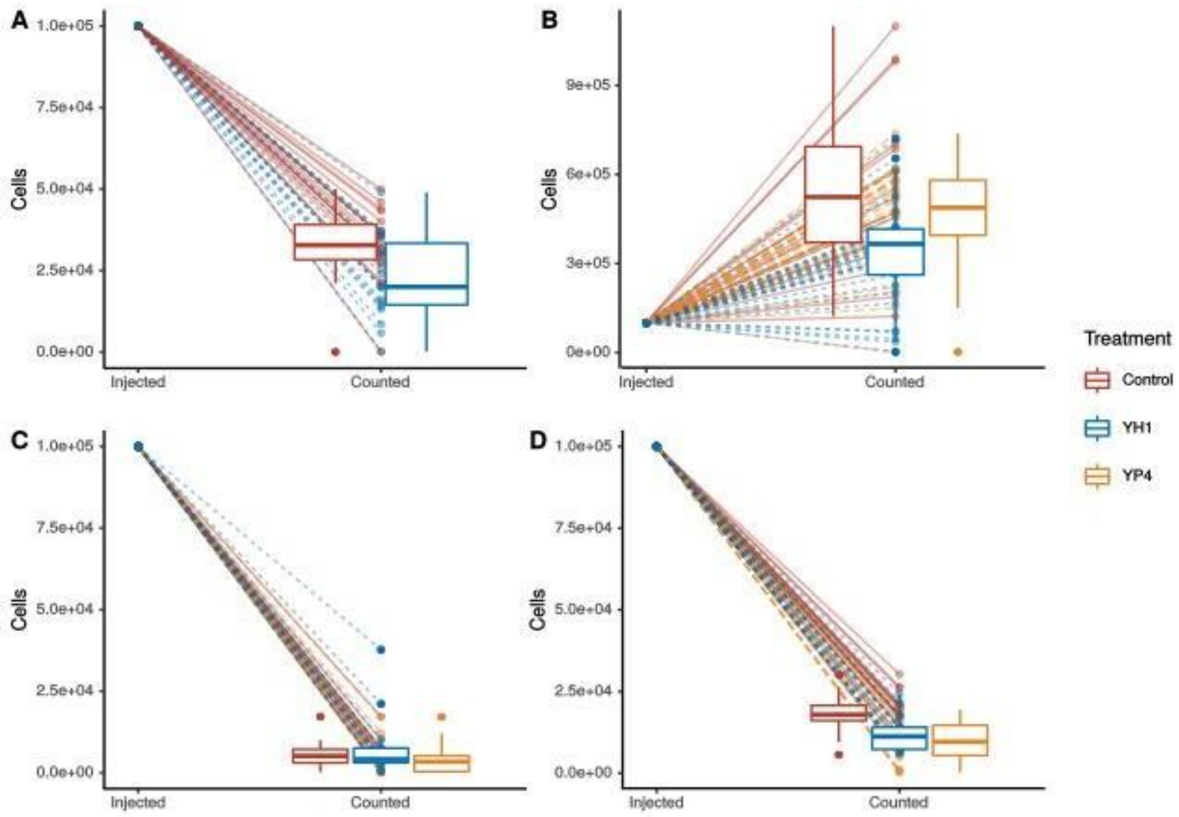

Supplementary Figure 4. Variation rate of bacterial clearance. Line charts and box plots showed the variation rate of *E. coli* cells represented on Y axis as a number of cells (cells) in both injected load (10^5 cells for infection) and residual counts (cells after 24 hours post-infection), reported on X axis (Injected and Counted) respectively. Bacterial clearance rates were showed considering the treatment groups such as control (red), YH1 (blue) and YP4 (orange) and divided *per* trials (A = Pre-diapause foundresses (Autumn 2016); B = Post-diapause foundresses (Spring 2017); C = Workers (Summer 2017); D = Pre-diapause foundresses (Autumn 2017)).

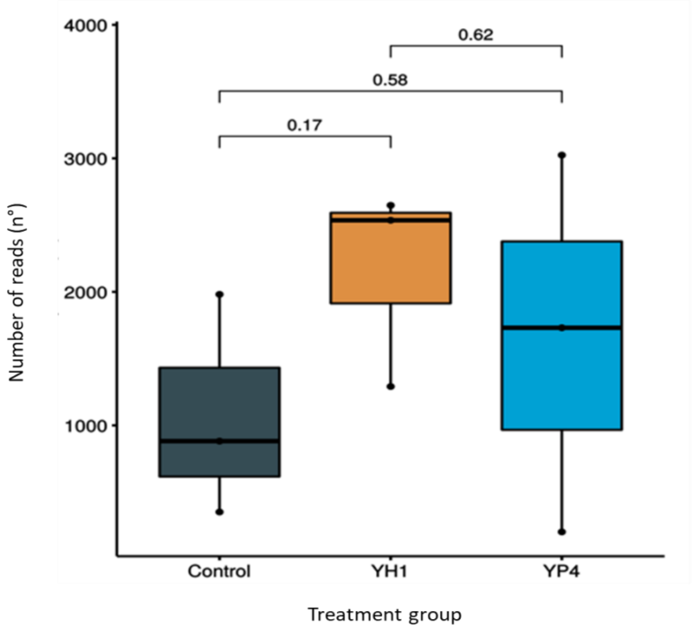

Supplementary Figure 5. Comparison of the number of sequence reads between control and treated groups. Boxplot showed the Number of reads (n°) per treatment group. P-values from t-test were reported above the box plot.

Datasheet S1. Generalized Linear Models (GLM) approach (*mvabund* package in R, negative binomial distribution) to assess the multivariate association between residual CFU/ml of *E. coli* injected (following the bacterial clearance) and gut bacterial communities composition.

Datasheet S2. Bacterial clearance: count of Colony Forming Units (CFUs) of injected *E. coli* after 24 hours from the infection.
